# Arteriovenous Malformation of the Vallecula Resulting in Recurrent Hemoptysis: A Case Report

**DOI:** 10.7759/cureus.84459

**Published:** 2025-05-20

**Authors:** Doha Amin, Kunal Matharu, Gabriella Manilla, Jatin Ahluwalia

**Affiliations:** 1 Otolaryngology - Head and Neck Surgery, Michigan State University, Detroit, USA; 2 Otolaryngology - Head and Neck Surgery, University of Toledo, Toledo, USA; 3 Otolaryngology - Head and Neck Surgery, Ascension Providence Hospital, Detroit, USA

**Keywords:** acute blood loss anemia, arteriovenous malformations, direct laryngoscopy, flexible fiberoptic laryngoscopy, recurrent hemoptysis, vallecula

## Abstract

Arteriovenous malformations (AVMs) of the upper airway in adults are relatively rare and can present with intermittent hemoptysis. Due to the intermittent nature of the bleeding episodes and frequent need for multidisciplinary interventions, diagnosing and treating upper airway AVMs can be challenging.

Here, a case of intermittent hemoptysis due to an AVM of the vallecula is described. It was identified using flexible laryngoscopy and was subsequently surgically removed with resolution of the patient’s symptoms. Overall, hemoptysis in adults has a broad differential and a thorough work up with multidisciplinary involvement should be performed.

## Introduction

Hemoptysis is a common cause of emergency department visits, with several thousand reported cases per year [1]. Common etiologies for hemoptysis in the adult population include bronchitis, pneumonia, bronchiectasis, gastrointestinal bleeding, upper airway malignancy, and pulmonary embolism [2]. Due to the broad differential diagnosis for hemoptysis, multidisciplinary intervention is commonly necessary. Most common interventions include radiography, computed tomography, laryngoscopy, esophagogastroduodenoscopy (EGD), and bronchoscopy [3]. While an obvious source is not identified in 20-50% of cases, a thorough work-up should be performed to avoid misdiagnosis [4]. 

Here, a case of an atypical etiology for hemoptysis in an adult patient is described. The patient was found to have an arteriovenous malformation of the right vallecula, resulting in recurrent hemoptysis and subsequent acute blood loss anemia. The lesion was identified with flexible laryngoscopy, and the patient underwent direct laryngoscopy in the operating room with complete en-bloc removal. Following the procedure, her hemoptysis resolved, and she was discharged in stable condition. Overall, accurately diagnosing and treating hemoptysis can pose challenges to clinicians due to the broad differential and frequent need for interdisciplinary involvement. Aerodigestive endoscopy and radiology are useful tools in the work-up for hemoptysis, and surgical intervention may be indicated for definitive treatment.

## Case presentation

The patient was a 54-year-old African American female with a past medical history of chronic obstructive pulmonary disease (COPD), hypertension, and active tobacco use, who presented to the emergency department with intermittent hemoptysis ongoing for 4 days. The patient stated she had unprovoked bleeding episodes every few hours. Although the exact volume could not be quantified, she estimated "several cups" worth of blood total. She was initially evaluated at an outside hospital two days before presentation. Hemoglobin upon arrival at the outside facility was 8.8 grams per deciliter (g/dL). She was evaluated by the gastroenterology and otolaryngology services at the outside facility. Esophagogastroduodenoscopy (EGD) and flexible laryngoscopy were both negative for active bleeding and unable to localize the site of bleeding. She was subsequently discharged.

**Figure 1:**
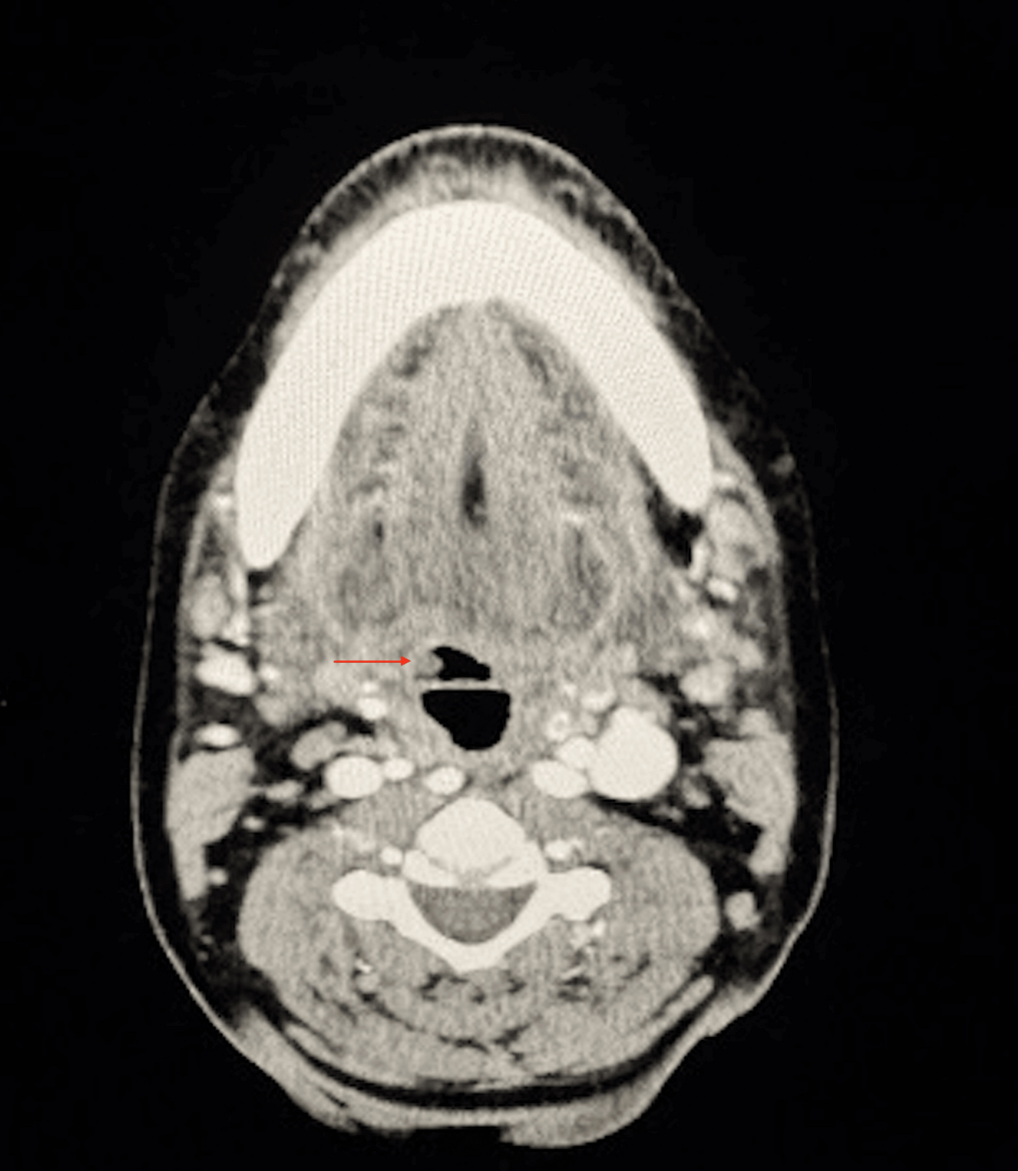
Axial CT image showing a small lesion arising from the right vallecula (red arrow).

Due to persistent hemoptysis, she presented to the authors’ facility two days after the initial evaluation. Upon arrival, her hemoglobin was found to be 5.6 g/dL. Evaluation by the otolaryngology service included repeat flexible laryngoscopy, which revealed pooled blood in the vallecula and piriform sinuses without localization. A subsequent EGD was performed by the gastroenterology service, which was negative for active upper GI bleeding; however, they did note a possible “bleb” along the lingual surface of the epiglottis, prompting re-evaluation by the otolaryngology service. Flexible laryngoscopy was again performed, which revealed an exophytic, circular purple lesion deep within the right vallecula. Both trans-nasal and trans-oral flexible laryngoscopy were performed at this time. The lesion bled upon both direct palpation with the flexible scope and with digital palpation of the tongue base. CT Angiography of the neck was performed and found to be negative for contrast extravasation (Figure 1). However, upon review of the imaging with the radiology service, there was a small lesion within the right vallecula. Subsequently, the decision was made to proceed to the operating room for further investigation. Differential diagnoses at this time included hemorrhagic cyst, hemangioma, vascular malformation, malignancy, vs other soft tissue lesions.

**Figure 2:**
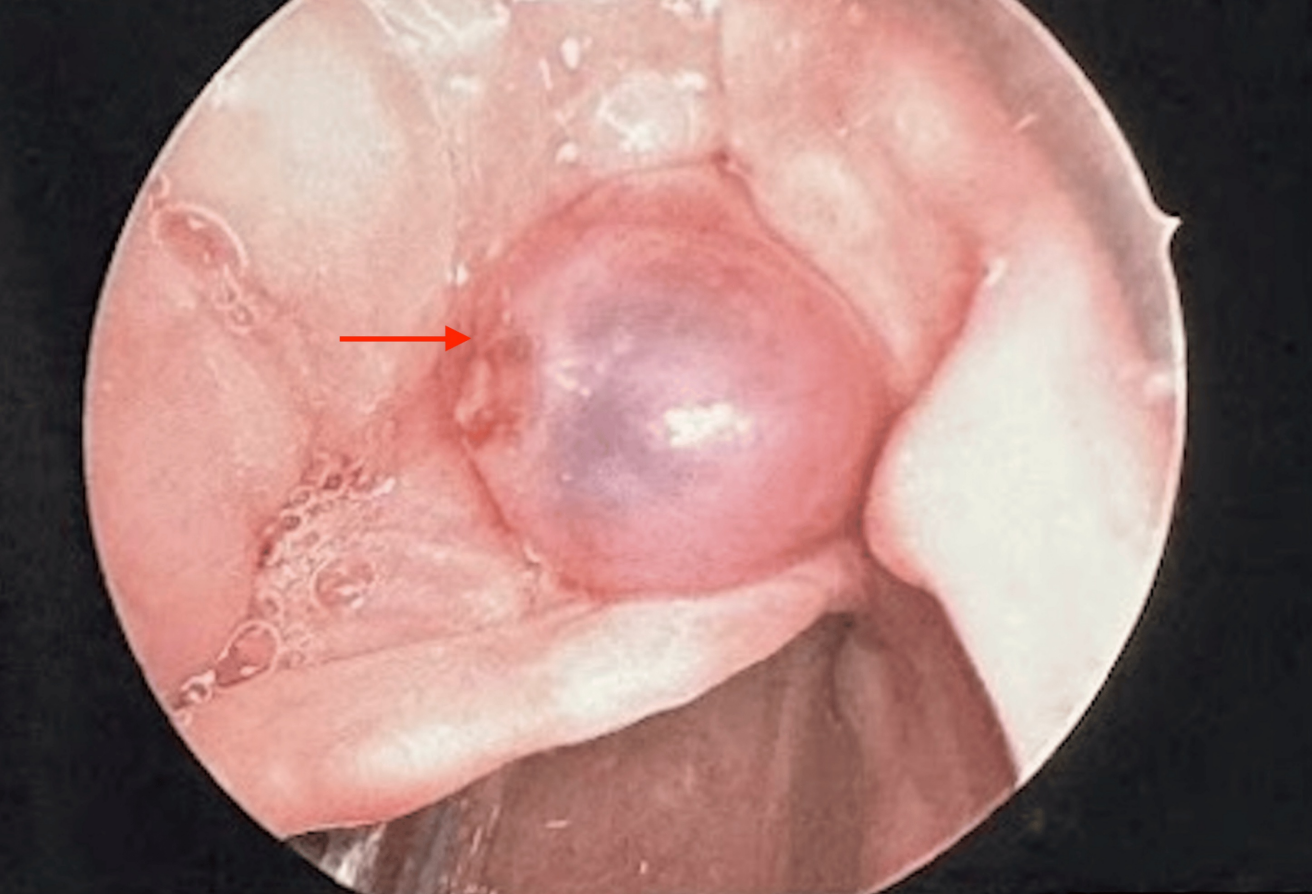
Intraoperative endoscopic images showing the vallecular arteriovenous malformation prior to surgical ablation (red arrow).

**Figure 3:**
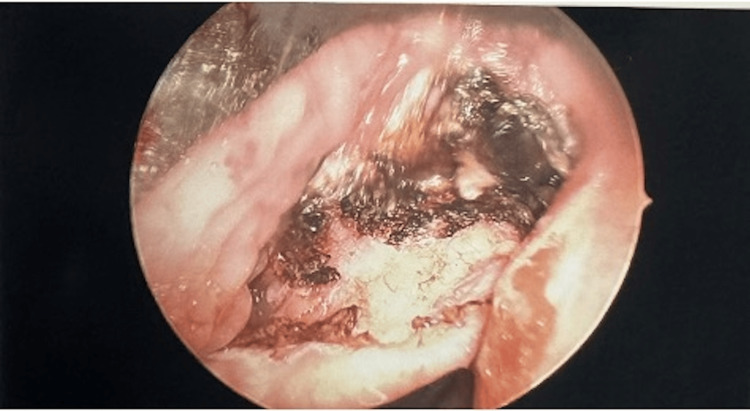
Intraoperative endoscopic image showing the arteriovenous malformation after removal and cauterization.

The decision was made to proceed with direct laryngoscopy, bronchoscopy, and possible biopsy with the otolaryngology service. The patient was brought to the operating room, and an uneventful orotracheal intubation was performed using video laryngoscopy. The lesion was noted within the right vallecula during intubation, and care was taken to avoid trauma. Once intubated, suspension microdirect laryngoscopy was performed. The laryngoscope was placed within the right vallecula, and the lesion was identified. A small area of trauma was noted on the medial aspect of the lesion, likely related to direct palpation from previous flexible laryngoscopy (Figure 2). The base of the lesion was cauterized with suction cautery, and the lesion was removed en bloc using up-biting cup forceps (Figure 3). There was no significant bleeding upon removal, and there was no evidence of large feeding vessels. The base of the lesion was then cauterized. There were several other small (<5 mm) vascular prominences noted within the base of the tongue, which were also cauterized. All additional subsites of the upper airway were visualized and found to be free of lesions. This included the oral cavity, oropharynx, hypopharynx, supraglottis, glottis, subglottis, trachea, and bilateral mainstem bronchi. Laryngotracheal anesthesia was then applied, and all instrumentation was removed. There was no further bleeding noted intra-operatively. 

The final pathology of the lesion was described as a “benign vascular lesion compatible with arteriovenous malformation”. It measured 2.4 x 0.3 x 0.3 cm in aggregate. 

The patient returned to the hospital floor post-operatively. She was started on a clear liquid diet and eventually advanced to full liquids without incident. In total, she received three units of packed red blood cells during her admission. Subsequent hemoglobin levels after the procedure were 7.8 g/dL and 8.1 g/dL. The patient was observed on post-operative day 1 and did not have further bleeding. A repeat laryngoscopy was not performed. The patient was then discharged on postoperative day 1 after having two stable hemoglobin levels and no further bleeding.

## Discussion

Hemoptysis in adults is caused by a wide variety of etiologies [1]. The severity can range from a self-limited event to a medical emergency. An accurate diagnosis relies upon a detailed history, appropriate imaging, as well as endoscopic and bronchoscopic evaluations [2]. Most commonly, hemoptysis in adults results from infections and malignancy of the pulmonary tract, with bronchiectasis (20%), lung cancer (19%), and bronchitis (18%) representing the most common cases [5]. Chronic bronchitis and bronchiectasis are more prevalent among patients with a history of tobacco use and COPD [5]. Although the most common causes stem from the lower respiratory tract, it is important to consider the additional less-common causes from the aerodigestive system, such as vascular malformations, upper respiratory tract neoplasms, and gastrointestinal (GI) bleeds. 

The vallecula refers to a small mucosal depression located at the base of the tongue, located between the folds of the throat on either side of the median glossoepiglottic fold [6]. This space serves an important physiological function: it entraps saliva, helping to prevent the premature initiation of the swallowing reflex, especially during sleep [6]. Embryologically, the vallecula is derived from ectoderm and develops from the third branchial arch [6]. Its innervation is supplied by the glossopharyngeal nerve (cranial nerve IX), which provides sensation in the oropharynx [6]. Vascular supply to the vallecular region comes from branches of the external carotid artery, particularly the facial and lingual arteries [6]. 

The differential for vallecular lesions is relatively broad, and they may also mimic AVMs. Vallecular hemangioma is a rare vascular tumor of the oropharynx, most commonly present in infancy or early childhood [7]. Extremely rare adult cases have been reported. Hemangiomas of the vallecula arise from the lingual surface of the epiglottis or adjacent tissues and are typically classified histologically as capillary or cavernous hemangiomas [7]. Symptoms vary by size and location but can include dysphagia, voice changes, foreign body sensation, hemoptysis, or airway obstruction in severe cases [8]. Additionally, lingual thyroglossal duct cysts (LTDCs) are rare congenital anomalies resulting from incomplete involution of the thyroglossal duct, with the base of the tongue being the most common ectopic location [9]. Vallecular LTDCs may present with dysphagia, dysphonia, airway obstruction, or feeding difficulties in infants and young children, though adult cases can also occur [10]. Finally, various malignancies can arise from the vallecula, including squamous cell carcinoma, myoepithelial carcinoma, and acinic cell carcinoma [11,12]. These lesions will typically appear irregular on laryngoscopy and can present with associated dysphagia, dysarthria, trismus, and cervical lymphadenopathy [11,12]. 

Upon our literature review, there have not been significant reports of vallecular AVMs presenting as repeated hemoptysis in the adult population. While upper airway AVMs are relatively uncommon, pulmonary AVMs have been documented as causes of hemoptysis [13]. In the case of this patient, the source was found to be an arteriovenous malformation (AVM) in the vallecula, which is a relatively uncommon location for this vascular malformation to occur [14]. AVMs are abnormal connections between veins and arteries that bypass the capillary system, and due to their aberrant configuration, are more prone to spontaneous hemorrhage compared to normal vascular structures [15]. These vascular malformations may be congenital, secondary to trauma, chronic inflammation, or from surgical intervention [15]. 

The workup for hemoptysis includes computed tomography imaging, laryngoscopy, bronchoscopy, and esophagogastroduodenoscopy (EGD). While panendoscopy is necessary to visualize the upper aerodigestive structures, its role in identifying AVMs can be limited due to patient tolerance and intermittent symptoms. Flexible laryngoscopy demonstrates a high level of diagnostic accuracy similar to that of direct laryngoscopy for visualizing laryngeal lesions [16]. EGD also has high diagnostic accuracy when localizing bleeds, but its use is limited to the upper GI tract. When localizing AVMs in the airway, CT angiography is the gold standard. Although data is sparse, due to the overall rarity of upper airway AVMs and the generally small size of feeding vessels to these lesions, emerging evidence has been highlighted for its application in the upper respiratory tract [17]. 

Overall, the differential diagnosis for vallecular lesions is broad, and these lesions should be considered for patients who present with hemoptysis. Multidisciplinary intervention and radiologic studies are often utilized in the workup for hemoptysis. While an obvious source may not always be identified, a thorough investigation is warranted to avoid misdiagnosis. This case presents a unique scenario, as the endoscopic images and pre-operative CT angiography were more suggestive of a possible cystic lesion, while final histopathology was consistent with an AVM. This finding reinforces the value of relying on histopathology in cases where pre-operative work-up is inconclusive. 

## Conclusions

Hemoptysis in adults can be a challenging entity to treat due to its broad differential diagnosis and frequent need for multidisciplinary intervention. It necessitates a thorough workup including radiography, computed tomography, and endoscopy of the upper airway and gastrointestinal tract. While a clear source may not always be identified, a thorough investigation should be performed to avoid misdiagnosis. 

In this case, an adult female was found to have an AVM of the vallecula, which caused recurrent episodes of hemoptysis. The patient had a complete resolution of symptoms following surgical excision. While rare, AVMs of the upper airway must be considered when working up patients for hemoptysis. If discovered, surgical exploration and resection can offer definitive treatment. In this case, the diagnosis of AVM was confirmed on histopathology, despite the atypical endoscopic and imaging findings. In cases where pre-operative work-up is inconclusive, relying on tissue histopathology is critical to determine the true etiology.
